# Primary Aortitis: A Diagnostic Mimic of Intramural Hematoma Presenting With Complete Atrioventricular Block

**DOI:** 10.1016/j.jaccas.2026.108008

**Published:** 2026-04-22

**Authors:** Julio Vázquez Reguera, Beatriz Aguiar Bermúdez, José Manuel Medina Suárez, Pedro Peña Ortega, Iñigo Rúa-Figueroa Fernández de Larrinoa, José Miguel Carricondo Martínez, Javier León Santana, Haridian Mendoza Lemes

**Affiliations:** a Department of Cardiology, Hospital Universitario Doctor Negrín, Las Palmas de Gran Canaria, Spain; b Department of Rheumatology, Hospital Universitario Doctor Negrín, Las Palmas de Gran Canaria, Spain

**Keywords:** aortic valve, bradycardia, computed tomography, imaging, positron emission tomography

## Abstract

**Background:**

Differential diagnosis between aortitis and intramural hematoma is challenging yet critical. Atypical presentations, including atrioventricular conduction disorders, should raise suspicion of aortitis.

**Case Summary:**

A 57-year-old man presented with dizziness, aortic regurgitation, and complete atrioventricular block. Computed tomography suggested intramural hematoma, whereas fluorine-18 fluorodeoxyglucose positron emission tomography-computed tomography proved pivotal for the differential diagnosis. Infectious and autoimmune workup was negative. Immunosuppressive therapy led to partial recovery of complete atrioventricular block, but permanent pacing was ultimately required. A diagnosis of primary aortitis was established.

**Discussion:**

Aortitis can display atypical imaging features and may affect the conduction system. To our knowledge, this is the first reported case of primary aortitis presenting with complete atrioventricular block exhibiting conduction improvement after immunosuppressive therapy.

**Take-Home Messages:**

Primary aortitis can mimic intramural hematoma and present with complete atrioventricular block. Multimodal imaging is essential to achieve diagnostic accuracy. Immunosuppression may lead to conduction recovery.

## Visual Summary

**Figure:**
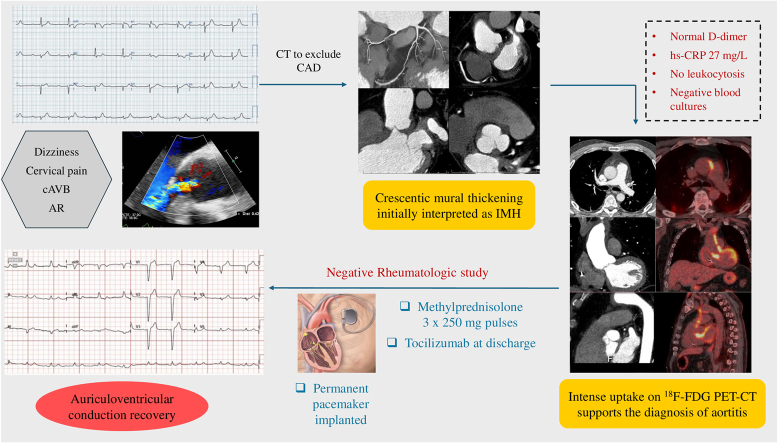

**Visual Summary:**
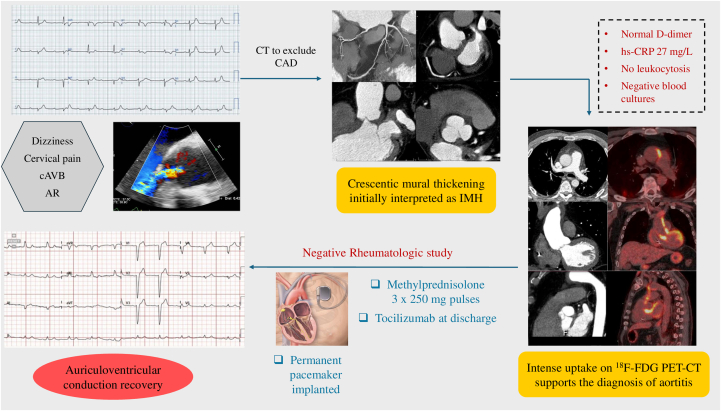
Clinical Timeline of Primary Aortitis Presenting With cAVB AR = aortic regurgitation; cAVB = complete auriculoventricular block; CAD = coronary artery disease; hs-CRP = high-sensitive C-reactive protein; CT = computed tomography; IMH = intramural hematoma; ^18^F-FDG PET-CT = fluorine-18 fluorodeoxyglucose positron emission tomography-computed tomography.

## History of Presentation

A 57-year-old man presented with 1-week history of dizziness and cervical pain.

**Take-Home Messages**

• Primary aortitis can mimic intramural hematoma and present with complete atrioventricular block.
• Multimodal imaging is essential to achieve diagnostic accuracy.
• Immunosuppression may lead to conduction recovery.

## Past Medical History

The patient was a smoker and had a history of hypertension, with no other relevant medical history.

## Physical Examination

On presentation, the patient was hemodynamically stable, without the need for supplemental oxygen. Cardiac auscultation revealed a grade III/VI early diastolic decrescendo murmur at the left sternal border. Lung examination was normal, with no peripheral edema or peripheral stigmata of infective endocarditis. Prior medical records showed no previously documented cardiac murmur, supporting this as a new-onset finding.

## Differential Diagnosis

In a young patient with a new-onset murmur and conduction disturbances, the first diagnosis to rule out is endocarditis. Other etiologies include acute aortic syndrome, aortitis (both infectious and noninfectious), or Lyme disease.

## Investigations

**Figure 1:**
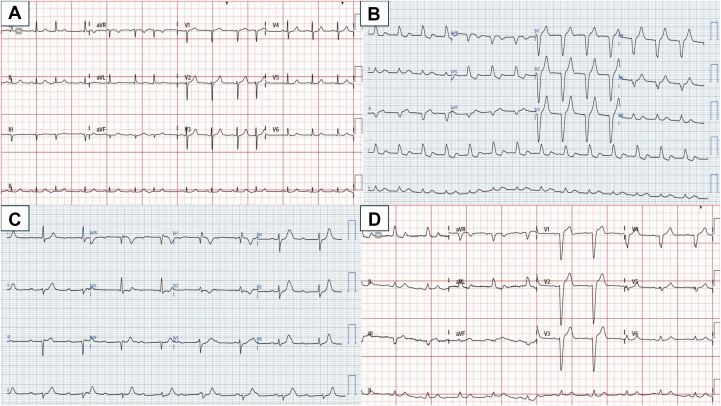
Evolution of the ECG From top to bottom and left to right: (A) Baseline ECG 1 year earlier; (B) on presentation to the emergency department, sinus rhythm with a PR interval of 340 ms and LBBB; (C) cAVB with a junctional escape rhythm; (D) partial recovery of atrioventricular conduction, with sinus rhythm, normalization of the PR interval, and persistent left bundle branch block. cAVB = complete atrioventricular block; ECG = electrocardiogram; LBBB = left bundle branch block.

**Figure 2:**
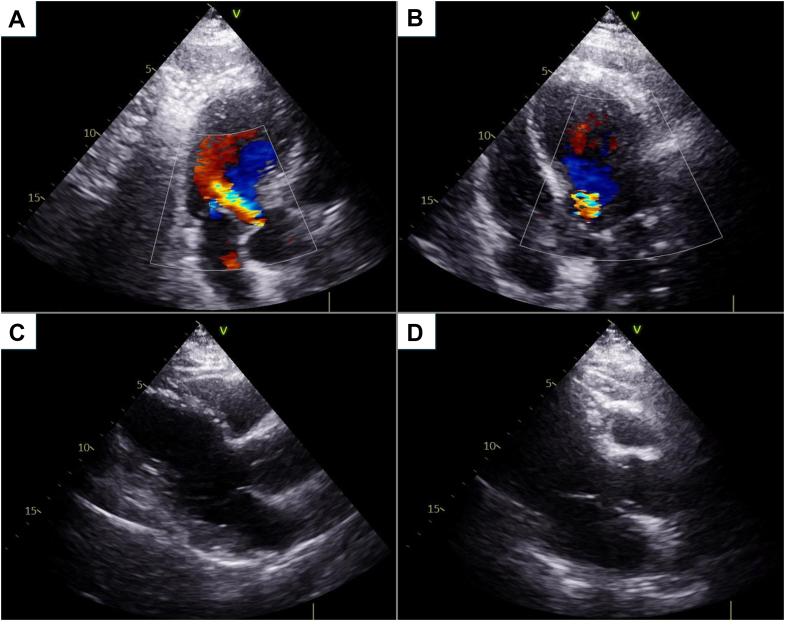
Baseline Echocardiogram Baseline echocardiogram showed grade III/IV AR (A and B) without evidence of an intimal flap (C and D). AR = aortic regurgitation.

The initial electrocardiogram showed sinus rhythm with a prolonged PR interval (340 milliseconds) and a previously undocumented left bundle branch block (LBBB), which progressed to complete atrioventricular block (cAVB) within an hour (Figure 1). The chest x-ray showed no significant findings. Laboratory tests demonstrated normal cardiac enzymes and N-terminal pro–B-type natriuretic peptide level of 1,954 ng/L. No leukocytosis was found. The baseline echocardiogram revealed grade III/IV aortic regurgitation (AR). No findings suggestive of an intimal flap were identified (Figure 2, Video 1). He was admitted to the cardiology department for further evaluation.

**Figure 3:**
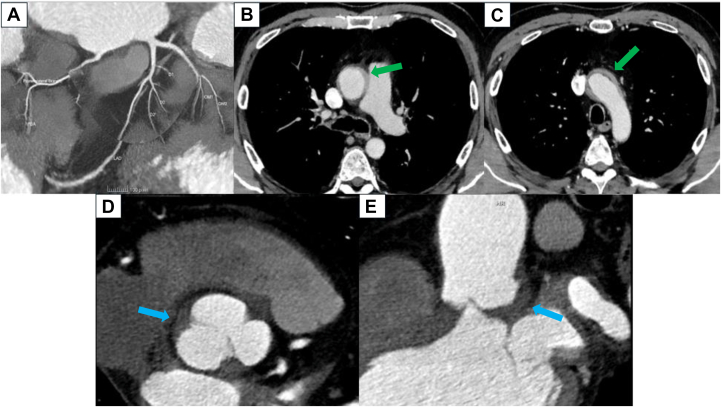
CT Scan (A) No coronary artery disease. (B and C, green arrows) Crescent-shaped mural thickening of approximately 7 mm along the aortic wall, extending from the root to the arch. (D and E, blue arrow) At the level of the annulus, the mural thickening extended into the aorto-mitral junction. CT = computed tomography.

Cardiac computed tomography (CT) ruled out coronary artery disease. Instead, it revealed a hyperdense, crescent-shaped intramural aortic thickening extending from the root to the distal arch, with involvement of the aorto-mitral junction. This was initially interpreted as an intramural hematoma (IMH) (Figure 3).

**Figure 4:**
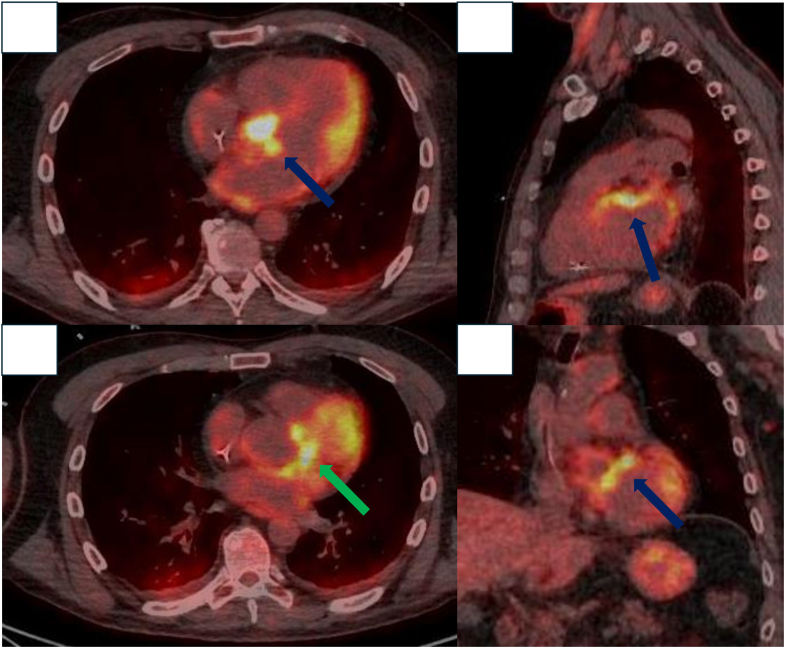
^18^F-FDG PET-CT Crescent-shaped intramural inflammatory uptake involving the aortic root, with involvement of the aorto-mitral junction. ^18^F-FDG PET-CT = fluorine-18 fluorodeoxyglucose positron emission tomography-computed tomography.

**Figure 5:**
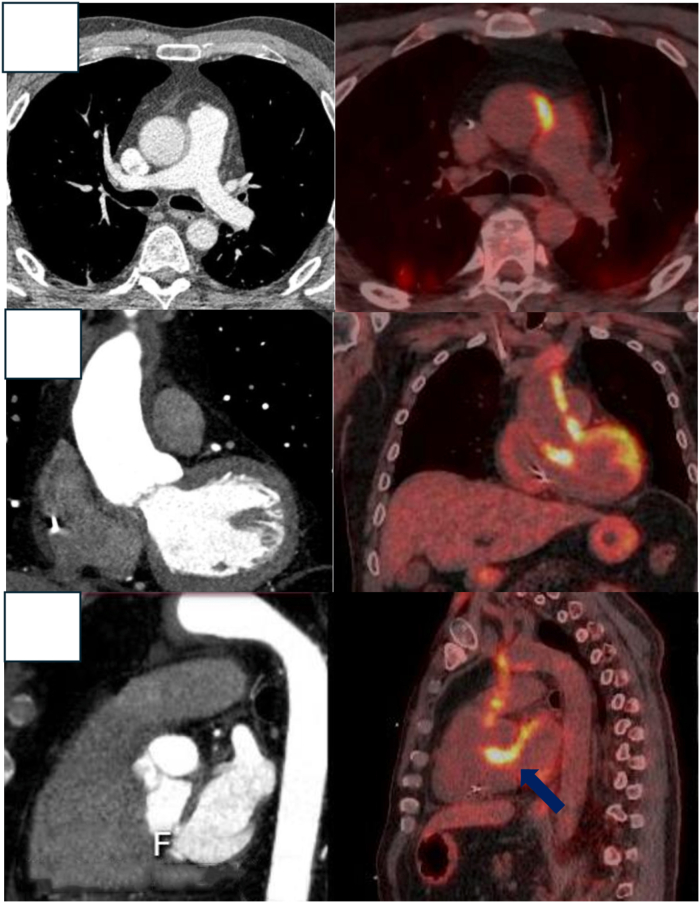
^18^F-FDG PET-CT Pathologic inflammatory uptake involving the wall of the ascending aorta, extending from its root to the proximal arch, with involvement of the aorto-mitral continuity (blue arrow). Transient pacemaker is also observed. ^18^F-FDG PET-CT = fluorine-18 fluorodeoxyglucose positron emission tomography-computed tomography.

Given the patient's hemodynamic stability and absence of symptoms, follow-up chest and abdominal CT scans were performed at days 7 and 15, showing no progression of the mural thickening. Subsequent fluorine-18 fluorodeoxyglucose positron emission tomography–CT (^18^F-FDG PET-CT) demonstrated markedly intense, heterogeneous uptake in the same region, including the aorto-mitral junction (Figures 4 and 5), with a maximal standardized uptake value of 11.57, exceeding blood pool activity (SUVmax 2.1) and hepatic uptake (SUVmax 3.3).

Additional laboratory tests assessing inflammatory markers revealed a C-reactive protein of 27 mg/L, with normal D-dimer. Infectious causes were excluded through negative blood cultures, Quantiferon testing, and targeted serologies. Rheumatologic evaluation—including serum protein electrophoresis, immunologic studies, and nailfold capillaroscopy—showed no significant abnormalities. Serum immunoglobin Ig4 (IgG4) levels were also not elevated. Cardiac magnetic resonance imaging was not performed due to patient claustrophobia. Biopsy was not undertaken because of the technical difficulty related to the location of the uptake.

**Figure 6:**
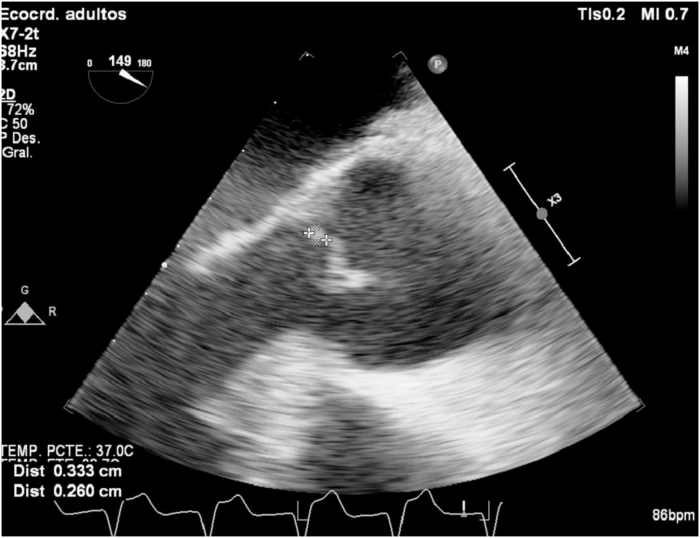
Lambl's Excrescence Mobile structure measuring approximately 3 mm on the ventricular aspect of the noncoronary cusp. This finding was interpreted as a Lambl's excrescence.

**Figure 7:**
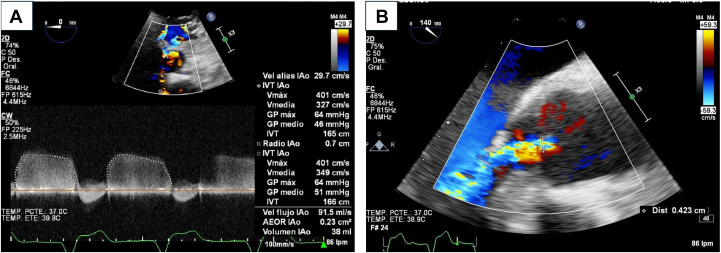
TEE and AR (A and B) Grade III/IV aortic regurgitation (effective regurgitant orifice area 0.23 cm^2^, regurgitant volume 38 mL, vena contracta 4.2 mm). AEOR IAo = effective regurgitant orifice; AR = aortic regurgitation; TEE = transesophageal echocardiogram; Volumen IAo = regurgitant volume.

Further evaluation was completed with a transesophageal echocardiogram (TEE). It revealed a trileaflet aortic valve with a filiform, mobile structure on the ventricular aspect of the noncoronary cusp. In the absence of fever, leukocytosis, repeatedly negative blood cultures, no uptake in ^18^F-FDG PET-CT, and follow-up TEE at 15 days showing no interval changes, this finding was interpreted as a Lambl's excrescence (Figure 6, Video 2). TEE confirmed grade III/IV AR (effective regurgitant orifice area 0.23 cm^2^, regurgitant volume 38 mL, vena contracta 4.2 mm) and a slightly dilated left ventricle (Figure 7, Videos 3 and 4).

## Management

The patient remained hemodynamically stable throughout the clinical course. Persistent cAVB finally required temporary pacemaker implantation. After the CT findings, the patient was transferred to the intensive care unit because of suspected aortic IMH.

**Figure 8:**
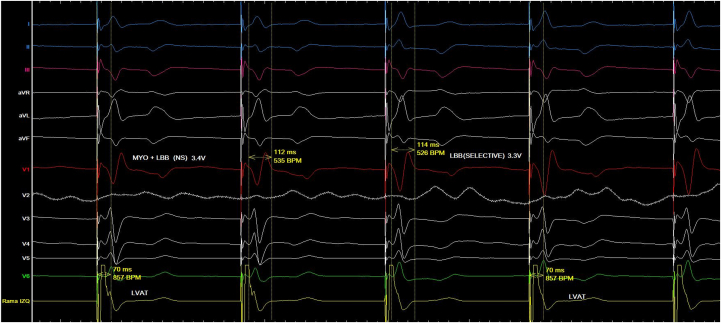
Differential Left Bundle Branch Capture Nonselective capture is observed at a pacing output of 3.4 V, and selective capture at 3.3 V, with an LVAT of 70 ms. LBB = left bundle branch; LVAT = left ventricular activation time; MYO = myocardial capture; NS = nonselective.

Based on the findings described earlier, the patient was ultimately diagnosed with idiopathic, noninfectious aortitis. In conjunction with the rheumatology team, immunosuppressive therapy was initiated. Intravenous methylprednisolone 250 mg daily for 3 consecutive days was administrated, followed by oral prednisone and methotrexate. After 7 days, intermittent recovery of atrioventricular conduction was observed. Given the absence of a defined etiology, risk of relapse despite immunosuppressive therapy, and lack of a sustained sinus rhythm after 14 days (alternating periods of sinus rhythm with a markedly prolonged PR interval and LBBB with episodes of atrioventricular conduction loss), a permanent pacemaker with physiological pacing was implanted (Figure 8).

Subcutaneous tocilizumab 162 mg weekly was initiated before hospital discharge. Additional medications included prednisone 40 mg daily (tapering planned) and antihypertensive therapy.

## Outcome and follow-up

**Figure 9:**
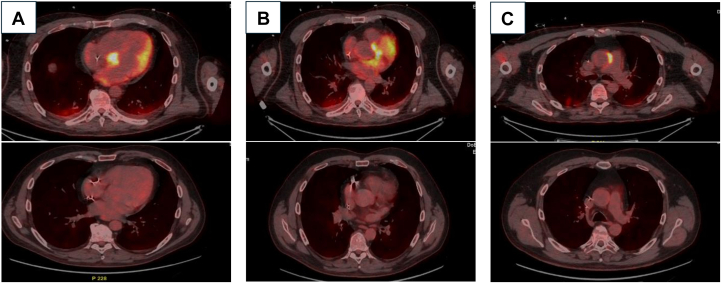
Comparison Between the Baseline ^18^F-FDG PET-CT (Top) and the Follow-Up Study Performed 1 Year After the Initiation of Immunosuppressive Therapy (Bottom) Normalization of the previously documented pathological uptake is observed at both the myocardial level (A) and the aorta (B and C).

The patient is in functional class I. In the last visit, he was in sinus rhythm with a PR interval of 200 milliseconds and LBBB, with <1% atrial pacing and 0% ventricular pacing. AR remains grade III/IV, with stable left ventricular volumes. Follow-up ^18^F-FDG PET-CT showed normalization of the previously observed pathological aortic and myocardial uptake (Figure 9). No new findings suggestive of an underlying etiology have been identified during follow-up.

## Discussion

Distinguishing between an aortic IMH and aortitis is challenging yet critical, as therapeutic implications differ markedly. Multimodality plays a central role by integrating morphological, metabolic, and functional information. Some authors have reported cases of aortitis mimicking IMH on imaging, presenting with aortic wall thickening but without true intramural bleeding.^1^^,^^2^

On CT, IMH is characterized by a hyperattenuating, crescentic thickening on noncontrast imaging and as a smooth, nonenhancing wall thickening after contrast administration. In contrast, aortitis usually shows circumferential, homogeneous aortic wall thickening and contrast enhancement, frequently accompanied by periaortic fat stranding.^3^^,^^4^ In our case, diagnosis was particularly challenging due to a crescent-shaped configuration, an uncommon but recognized presentation of early-stage aortitis, more frequently described in IgG4-related disease.^5^

Beyond classic large-vessel vasculitides such as Takayasu arteritis or giant cell arteritis, idiopathic, noninfectious (clinically isolated) aortitis should be considered. This entity is characterized by localized aortic inflammation without systemic vasculitis or infection. Imaging findings in clinically isolated aortitis may overlap with both IMH and systemic aortitis, typically showing focal or segmental wall thickening with variable enhancement. Unlike large-vessel vasculitides, it usually lacks diffuse vascular involvement, constitutional symptoms, or progressive luminal stenosis. Management is often individualized rather than protocol-driven.

In equivocal cases, ^18^F-FDG PET-CT plays a pivotal role.^3^ Recognition of inflammatory extension to adjacent structures is particularly relevant, as involvement of the aorto-mitral junction is an exceptionally rare manifestation of primary aortitis and may explain associated conduction disturbances such as cAVB. However, ^18^F-FDG PET-CT findings must be interpreted cautiously. First, FDG uptake may be false-negative or equivocal, particularly in early, low-grade, or partially resolving inflammatory stages. Second, clinically isolated aortitis may present with focal, heterogeneous, or segmental FDG uptake, reflecting a more localized inflammatory process. Finally, the ^18^F-FDG PET-CT imaging findings may represent partially resolving or chronic inflammation, with reduced metabolic activity despite persistent structural involvement on CT. These limitations underscore the need to integrate metabolic imaging with anatomical findings and clinical context.

Mild myocardial FDG uptake was observed (Figures 5 and 6) and interpreted as physiological. Cardiac sarcoidosis was considered. However, the myocardial signal was diffuse, without a typical patchy pattern; there were no extracardiac involvement, and serum calcium and angiotensin-converting enzyme levels were within normal ranges, further arguing against sarcoidosis.

**Figure 10:**
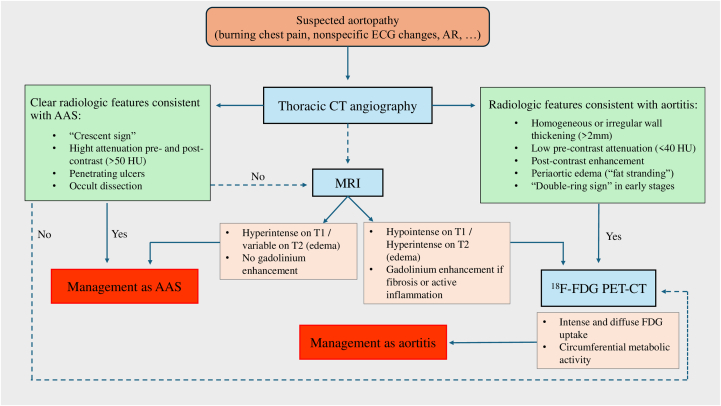
Differential Diagnostic Algorithm for Aortitis vs Acute Aortic Syndrome Multimodality imaging is key for differentiating aortic IMH from aortitis. AAS = acute aortic syndrome; AR = aortic regurgitation; CT = computed tomography; ECG = electrocardiogram; ^18^F-FDG PET-CT = fluorine-18 fluorodeoxyglucose positron emission tomography-computed tomography; HU = Hounsfield units; IMH = intramural hematoma; MRI = magnetic resonance imaging.

Cardiac magnetic resonance typically shows generalized wall thickening and inflammation with contrast enhancement in aortitis. In IMH, dynamic and contrast-enhanced sequences reveal patent flow in the main lumen, and the hematoma's appearance on T1- and T2-weighted sequences changes over time as blood clots age^4^ (Figure 10).

Laboratory markers are nonspecific in aortitis and may be normal, as can acute-phase reactants in IMH. IgG4-related aortitis is not consistently associated with elevated serum Ig4 levels.^6^ Biopsy, although diagnostic, is often not feasible due to anatomical constraints.

As we know, no case has been reported associating primary aortitis and cAVB although isolated reports of noninfectious vasculitis presenting with AVB have been described.^7^ A transient AVB has been noted in a case of post-COVID-19 aortitis.^8^ An additional IgG4-related aortitis case presented with cAVB demonstrated conduction recovery after high-dose methylprednisolone.^9^ In our report, recovery of atrioventricular conduction and shortening of the PR interval after treatment support an inflammatory cause of the cAVB. To our knowledge, this is the first reported case of idiopathic aortitis-related cAVB reversing with immunosuppressive therapy. cAVB may not be only a complication of aortitis but also an inflammatory marker, potentially reversible with immunosuppressive therapy. Nevertheless, given the time required for resolution of cAVB and the risk of clinal flares, permanent pacemaker implantation might be necessary in these patients. This clinical pattern—limited aortitis without aneurysm and cAVB—is a very unusual phenotype and underscores the importance of considering primary aortitis in young patients with new conduction disturbances.

Escalation to the interleukin-6 receptor antagonist tocilizumab was guided by imaging evidence of active disease, high-risk anatomical involvement, and severe conduction disturbance attributable to inflammatory involvement of the aorto-mitral junction given its temporal association with imaging findings, the absence of ischemic, infectious, or degenerative causes. Given the absence of alternative etiologies and concern for progression, biologic therapy was initiated to achieve durable inflammatory control and mitigate further structural or electrical complications.^10^

## Conclusions

Primary aortitis diagnosis is challenging, requiring a multimodal and multidisciplinary approach. Crescent-shaped aortic wall thickening should prompt consideration of aortitis in atypical presentations, as illustrated by this case. This report highlights the importance of considering this diagnosis in patients with suspected aortic syndromes accompanied by significant conduction disturbances.

## Funding Support and Author Disclosures

The authors have reported that they have no relationships relevant to the contents of this paper to disclose.

## Appendix

Video 1 Baseline Echocardiogram Baseline echocardiogram showed no evidence of an intimal flap.

Video 2 Lambl's Excrescence Mobile structure on the ventricular aspect of the noncoronary cusp, interpreted as a Lambl's excrescence.

Video 3 Transesophageal Echocardiogram TEE showed grade III/IV AR. AR = aortic regurgitation; TEE = transesophageal echocardiogram.

Video 4 Transesophageal Echocardiogram TEE showed grade III/IV AR. AR = aortic regurgitation; TEE = transesophageal echocardiogram.
